# Transcript expression profiling in two contrasting cultivars and molecular cloning of a SKP-1 like gene, a component of SCF-ubiquitin proteasome system from mungbean *Vigna radiate* L.

**DOI:** 10.1038/s41598-019-44034-4

**Published:** 2019-05-30

**Authors:** Nandita Bharadwaj, Sharmistha Barthakur, Akash Deep Biswas, Monoj Kumar Das, Manpreet Kour, Anand Ramteke, Nirmali Gogoi

**Affiliations:** 1 Department of Environmental Science, Tezpur University, Tezpur, 784028 Assam India; 2 ICAR-National Research Centre on Plant Biotechnology, Pusa Campus, New Delhi, 110012 India; 3 Department of Chemistry, Scuola Normale Superiore di Pisa, Piazza dei Cavalieri, 7, Pisa, 56126 Italy; 4 Department of Molecular Biology and Biotechnology, Tezpur University, Tezpur, Assam 784028 India

**Keywords:** Computational biology and bioinformatics, Genetics, Molecular biology, Physiology

## Abstract

Protein degradation and turnover under various environmental stresses is basically regulated by ubiquitin-proteasome system (UPS), of which SKP1 is a very essential component. Isolation and cloning of an identified potential stress responsive candidate gene SKP1, was successfully done for the first time to fathom the role of SKP1 in drought tolerance at genetic level in drought tolerant mungbean cultivar Pratap, which was screened after a detailed physio-biochemical screening amongst seven popular mungbean cultivars. The cloned gene SKP1 (accession number KX881912) is 550 bp in length, encodes 114 amino acids. It shows high sequence homology with SKP1 from *Zea mays* (NP_001148633). The protein expression of isolated SKP1 was confirmed by GUS fused expression using a Histochemical assay under control as well as under drought stress. Further, up-regulation in relative expression level of SKP1 in different plant parts under drought stress confirmed its utility as a potential drought responsive candidate gene certainly demanding extensive genetic research for further incorporation in breeding programs. Moreover, the structure of VrSKP1 (*Vigna radiata* SKP1) has been modelled, validated and an Essential Dynamics (ED) was done on the Molecular Dynamics (MD) simulation trajectories for filtering large-scale concerted motions. Free-energy calculations on the ED revealed a complex free-energy landscape (FEL) implying the conformational diversity of the modelled VrSPK1 protein.

## Introduction

Climate change has emerged as a major concern throughout the world threatening crop productivity and drought is considered as one of the most potential devastating events responsible for reduction in crop yield^1^. Food security and sustainability being a prime concern today, study of plants in both physiological and molecular grounds has become quintessential. This, in effect, necessitates adopting genetically improved cultivars with better stress tolerance.

Mungbean is an important tropical short duration grain legume. It is a principal cash crop with high protein content and nitrogen fixing ability. Mungbean production has been greatly threatened with the expanding drought stressed area. Genetic and molecular information of mungbean when compared to other legumes is still lacking and requires attention and in-depth research. This is essential for breeders to develop drought-tolerant cultivars which would survive stress better without much damage to the plant system. A better understanding of the underlying physiological processes is mandatory to screen a tolerant cultivar from the rest. Blending of physiology and genetic analyses is therefore necessary towards identification of tolerant cultivars which can serve as a source of candidate genes and can further be manipulated to develop tolerant germplasm that are able to cope in the changing climate scenario.

Candidate genes which have been explored till date in legumes are involved in combating stress by augmenting the level of compatible solutes like proline, starch, sugars etc.^2^. This, in turn, shields the plant by cellular osmotic adjustment, detoxification of ROS, protection of membrane integrity and stabilization of enzymes/proteins^3,4^. Several drought stress responsive candidate genes have been reported in different grain legumes such as MYB^5^, ZIP^6^, and DREB2A^7^ from Chickpea, DREB2B^8^ in common bean, DREB, NAC, and ZIP, in soya bean^9^, CPRD8, CPRD12, CPRD14, CPRD22^10^ in Cowpea, CcM1522–CcM1821, and CcM0047–CcM2332 in Pigeon pea^11^ etc. However, isolation of drought tolerant candidate gene from mungbean hasn’t been reported till date^12^.

SKP1 (S-phase kinase-associated protein 1) gene is involved in proteolysis^13^ and plays crucial role in stress tolerance^14,15^. SKP1 protein is a core component of SCF [SKP1-RBX1-CUL1-F-box protein (SCF)] E3 ubiquitin ligase complex, an adapter protein which interacts with cullin and F-box proteins. SCF complexes are known to play regulatory role in signalling pathways of various phytohormones namely ABA, Auxin, Jasmonic acid, Brassino steroid, Gibberellins, and Ethylene^16^. *Arabidopsis* SKP1-like protein (ASK1) is the most well studied SKP1 till date. SKP1 genes from *Solanum pimpinellifolium*, SSK1 and SSK2 genes, functionally similar to ASK1^17^, SKP1 from common bean^18^, wheat^1^ and cotton^13^ had also been reported.

GUS assay^19^ has been used as a quick screening method to check the transient expression of the recombinant gene in the putative transformed explants. Although, F-box protein genes, another component SCF ligases, contain heat, drought, SA, ABA, elements and respond quite positively to abiotic stresses^20^, less is known about SKP1 and its role in abiotic stress tolerance. Very recently, a homologue of SKP1, from *Paeoniasuff ruticosa* rendered salinity tolerance when overexpressed in *Arabidopsis*^21^. Over-expression of GsSKP21 from *Glycine soja* in Arabidopsis showed ABA sensitivity and improved tolerance to alkalinity^22^. The objective of this study was to carry out transcript expression profiling of SKP1 at vegetative stage in different plant parts of drought-tolerant (Pratap) and drought-susceptible (IPM 99-125) mungbean cultivars under water stress and to isolate a full length SKP1 gene and further screen the transient gene expression by GUS assay.

Survival and functioning of an organism is based on the role of proteins, generally determined by their three-dimensional (3D) structures. Therefore, deduction of its 3D structure from its primary structure has been considered as one of the major goals to understand the structural dynamics of the protein. Proteins are known to be dynamic entities in cellular solution. The dynamic personalities of the protein decide its functional properties. In this study, we made an effort to predict and report the 3D structure of the VrSKP1 protein and study various properties of the same. The VrSKP1 protein’s 3D structure is not yet solved experimentally so it has been predicted by an *in-silico* approach and the structural insight has been reported. Thus, we aimed to study the MD simulation followed by validation of the structure and its stability. Trajectories generated from MD simulations provide a means to identify and study motions that are crucial for protein functions. Separating functionally important motions is a major challenge in analysing MD trajectories. So, to study the differences in the dynamical motions of the VrSKP1 protein an ED was also done on the trajectories of MD simulation to separate large-scale correlated motions from local harmonic fluctuations. The free-energy landscape that is very rugged with multiple minima and transition states, was further performed to achieve a remarkable efficiency in determining the stable structure of the VrSKP1 modelled protein.

## Results

### Effects of water stress on biochemical traits of mungbean cultivars

**Figure 1:**
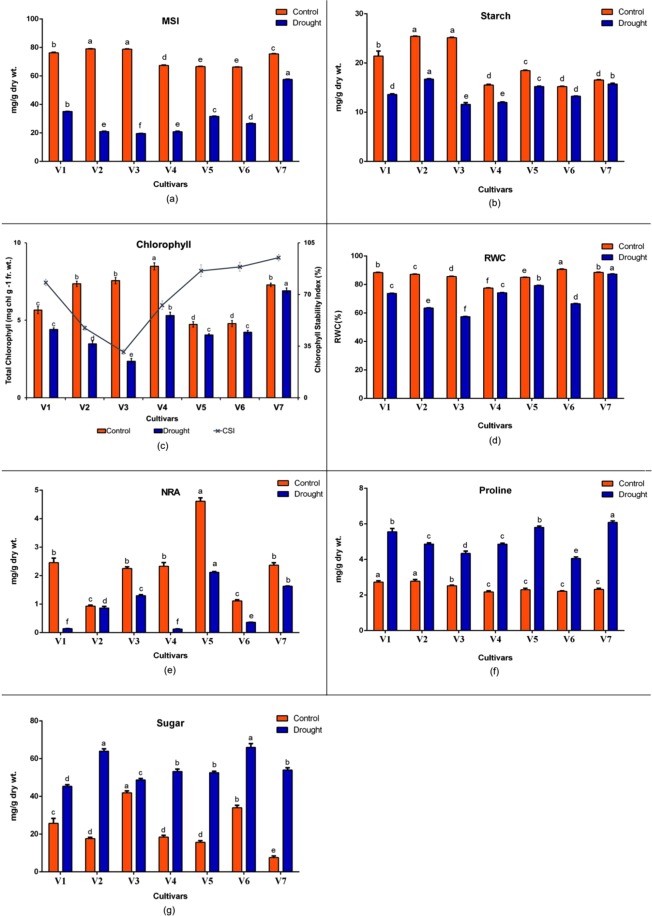
Effect of drought stress on membrane stability index (**a**), starch (**b**), leaf chlorophyll content (**c**), RWC (**d**), nitrate reductase activity (**e**), proline (**f**) and sugar (**g**) in seven mungbean varieties [PDM54 (V1), PDM39 (V2), IPM 99-125 (V3), PDM11 (V4), IPM 2-14 (V5), IPM 2-3 (V6) and Pratap (V7)] on 21^st^ day for ten consecutive days. Mean sharing the same letters, for a parameter in different cultivars; don’t differ significantly at p ≤ 0.05.

Water stress resulted in statistically significant (p ≤ 0.05) changes in the studied biochemical indices (Fig. 1a–g). Drought stress reduced MSI significantly irrespective of the cultivars considered. Though all the mungbean cultivars didn’t show significant differences under control, they behaved considerably different when water stress was imposed. Highest fold reduction of MSI was noted in V3 (IPM 99-125) (75%) followed by V2 (PDM39), V1 (PDM 54) and V4 (PDM11). On the contrary, V7 (Pratap) recorded the least MSI reduction (24%).

Similar trend was seen with significant decrease in starch content in all the seven cultivars after stress for ten consecutive days. Differences in starch content were found to be significant (p ≤ 0.05) in the cultivars V2 (PDM 39) and V3 (IPM 99-125) under drought, whereas they responded alike under control. On the other hand, V3 (IPM 99-125) and V7 (Pratap) responded quite significantly (p ≤ 0.05) different both under control and drought which also corresponds to their highest (54%) and the lowest (5%) percentage decrease in starch levels respectively.

Lower total chlorophyll content was observed in all cultivars after treatment. Recorded values indicated significant (p ≤ 0.05) differences under water stress between V2 (PDM39), V3 (IPM 99-125) and V7 (Pratap) which contrasted with observations under control. Whereas, V1 (PDM54) and V6 (IPM 2-3), which were similar under control, responded significantly different under drought. Furthermore, V3 (IPM 99-125) was seen to show its maximum susceptibility with highest percentage decrease (68%) of total chlorophyll content under stress. Adding and reconfirming the former results, sharp drop in CSI was noted in V3 (IPM 99-125), while the highest CSI was observed in cultivar V7 (Pratap).

NRA was also recorded to have significant (p ≤ 0.05) reduction for all the genotypes under water stress. Genotypes V1 (PDM54), V3 (IPM 99-125), V4 (PDM11) and V7 (Pratap) showed significant (p ≤ 0.05) differences when watering was withdrawn, though they were all seen to be similar in control. A similar pattern of NRA was also observed in V2 (PDM39) and V6 (IPM 2-3) under water stress. However, the highest and the lowest percentage decrease in NRA activity after drought was seen in V4 (94.5%) and V2 (7.2%) respectively.

A significant (p ≤ 0.05) decrease in RWC of leaves was recorded for all the cultivars under drought while, the highest percentage reduction (54%) was seen in V3 (IPM 99-125). Significant differences (p ≤ 0.05) were recorded between the cultivars under control and drought. Water stress significantly increased proline content in all the cultivars. Statistical analysis demonstrated higher significant (p ≤ 0.05) differences among the cultivars and also within the treatments. Cultivars V4 (PDM11), V5 (IPM 2-14), V6 (IPM 2-3) and V7 (Pratap) though behaved in a similar manner under controlled condition, they showed significantly (p ≤ 0.05) different response under drought. However, V3 (IPM 99-125) and V7 (Pratap) have shown significantly different behaviour both under controlled and drought stress conditions. Coinciding their differences, cultivar V7 (Pratap) responded more sharply for accumulating highest proline content (62%), while the least value (42%) of proline production was recorded in cultivar V3 (IPM 99-125).

Similarly, sugar accumulation was also found to increase in all the cultivars significantly (p ≤ 0.05) after drought stress. Differences in sugar accumulation in genotypes V1 (PDM 54), V3 (IPM 99-125) and V7 (Pratap) were significant (p ≤ 0.05) both after and before stress. Whereas, cultivars V4 (PDM11) and V5 (IPM 2-14) behaved quite similarly under both the conditions with non-significant differences between the two. However, cultivars V2 (PDM39) and V6 (IPM 2-3) have shown significant (p ≤ 0.05) differences in control though they behaved similarly during drought. Cultivar V3 (IPM 99-125) was found to show the least (14%) percentage change of sugar production under water stress, whereas cultivar V7 (Pratap) showed the highest (86%).

Therefore, with significant differences (p ≤ 0.05) between the cultivars Pratap (V7) and IPM 99-125 (V3) for sugar, proline, MSI, starch, RWC under control as well as drought and for NRA and total chlorophyll content only under drought, it becomes quite evident that both the cultivars respond differentially under drought stress (Table 1). Moreover, a significant (p ≤ 0.05) percentage increase/decrease in their performance for all the studied parameters except NRA reveals V7 (Pratap) to be the most drought tolerant cultivar, while cultivar V3 (IPM 99-125) the most susceptible.

**Table 1:** The mean square values of the treatments (T), cultivars (V) and the treatment–cultivar interaction (T × V) along with their errors and significance.

Sources of variation	Parameters						
	MSI	Starch	Chlorophyll	RWC	NR	Proline	Sugar
Treatment (T)	24711.56**	438.80**	9.61**	2896.30**	6.35**	95.46**	369.29**
Cultivar (V)	407.92**	50.08**	65.14**	227.33**	25.94**	1.25**	14173.74**
T × V	376.19**	40.97**	7.34**	248.96**	1.83**	1.13**	411.02**
Error	0.124	0.279	0.092	0.098	0.016	0.024	4.916

**Significant at 0.01 probability level.

### Effects of water stress on SKP1 gene expression

**Figure 2:**
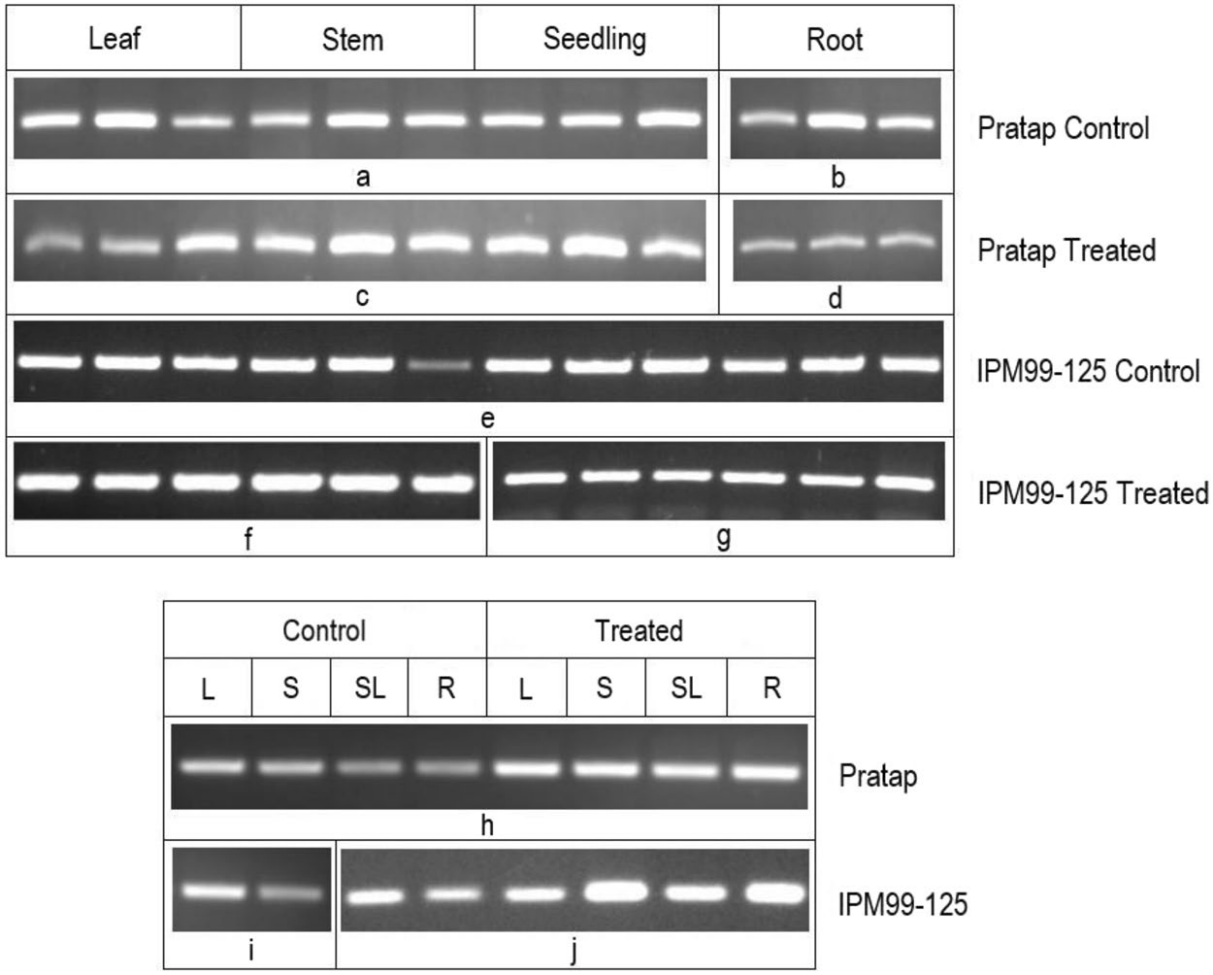
Semi Quantitative RT-PCR of SKP1 transcripts after drought stress on replicates of different plant tissues during vegetative stage of the plant in two diverse mungbean ideotypes, Pratap and IPM 99-125 where; L: Leaf tissue, S: Stem tissue, SL: Seedling tissue and R: Root tissue (**a**) and (**b**) representing Lane 2–10 from Gel picture A and Lane 3–5 from Gel picture B respectively (**c**,**d**) representing Lane 2–10 from Gel picture C and Lane 8–10 from Gel picture B (**e**) representing Lane 2–13 from upper stack of Gel picture D (**f**,**g**) representing Lane 2–7 and 9–14 respectively from lower stack of Gel picture D (**h**) representing Lane 2–9 from Gel picture E and (**i**,**j**) representing Lane 10–11 from Gel picture E and Lane 2–7 from Gel picture F respectively for actin (Full length gels are presented in Supplementary File 1).

Towards transcriptional profiling of SKP1 under drought condition in various tissues in mungbean, relative expression levels of SKP1 was studied in representative susceptible V3 (IPM 99-125) and tolerant V7 (Pratap) cultivars after screened on the basis of seven physio-biochemical indices (Fig. 2). The primers used in the study (Table 2) were designed from available gene sequences in the NCBI database. Comparisons were made within and between the cultivars with and without water stress treatment. SKP1 transcripts were found to be differentially expressed, in both the cultivars irrespective of the treatments and even under controlled conditions.

**Table 2:** Primers used in the study.

Primer Name	Primer sequence	Primer Purpose
Actin forward	5′AGCGAGTCTTCATAGGGCGAT3′	Amplification of actin expression
Actin reverse	5′TAGCTCTGGGTTCGAGTGGCA3′	Amplification of actin expression
SKP1 (exp) forward	5′TGGCTGCCAACTACCTGAACA3′	cDNA amplification and relative expression
SKP1 (exp) reverse	5′ACATGTTCACCGACACCACCT3′	cDNA amplification and relative expression
SKP1 (full) forward	5′ACCACTGACCGAAGATGTTCACCA3′	cDNA amplification and cloning
SKP1 (full) reverse	5′GAGCGATGGCGGCCGCGGGAGAC3′	cDNA amplification and cloning
SKP121XbF	5′ GGTCTAGAATGGCGGCCGCGGGAG 3′	Amplification of coding transgene region (*xbaI site underlined)*
SKP121BmR	5′CGCGGATCCCTACTCAAAGGCCCTCTGG 3′	Amplification of coding transgene region (BamH1 site underlined)

Within the cultivars, SKP1 was highly up-regulated in the leaves (25%) and stem (39%) of the tolerant Pratap under water stress, while the roots (31%) and whole seedling (25%) recorded a statistically significant (p ≤ 0.05) down-regulation. The sensitive variety on the contrary showed an up-regulation of SKP1 under drought with a percentage change of 57%, 62%, 48%, and 56% in leaf, stem, root and whole seedling respectively.

**Figure 3:**
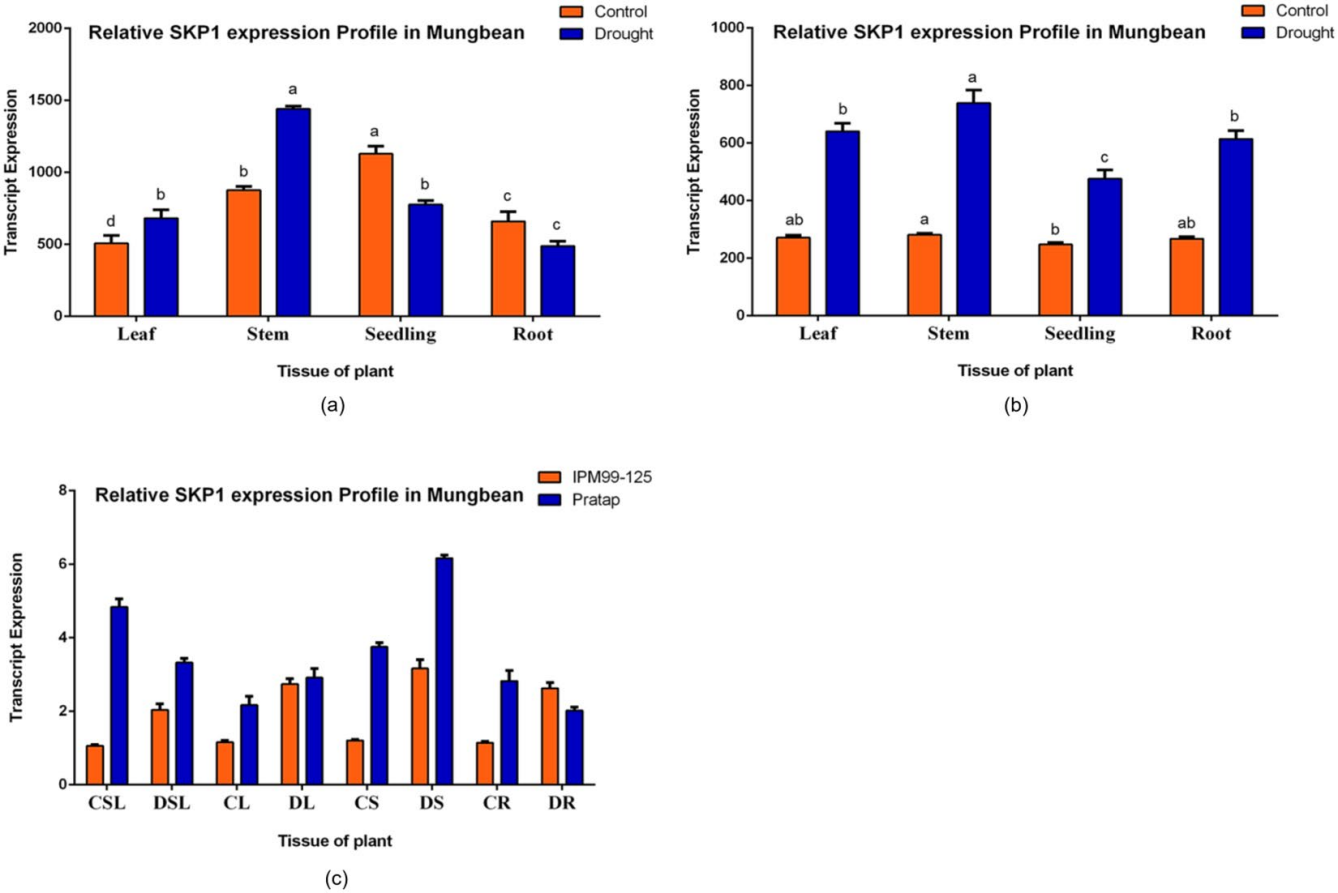
Comparative transcript expression profiling of SKP1 for drought tolerance (Mean ± S.E.) in two contrasting mungbean cultivars, Pratap (**a**) and IPM 99-125 (**b**) in vegetative stage of the plant while water was withdrawn for 10 consecutive days (considering absolute values). For relative quantification, least expression value from control whole seedling in the sensitive cultivar was taken as 1 and relative fold changes in expression were calculated for the remaining cultivar and the remaining plant tissues (**c**). Mean sharing the same letters for a parameter in the cultivars, don’t differ significantly at p ≤ 0.05.

The relative expression levels were then compared between the contrasting cultivars under water stress (Fig. 3). Under controlled conditions, significant (p ≤ 0.05) higher SKP1 transcript levels were recorded in the tolerant variety for most of the comparisons made, with a 78% of maximum increase in the whole seedling. Whereas under drought stress, stem, root and whole seedling showed significant SKP1 up-regulation; a statistically non-significant up-regulation was noted in the leaves and 20% down-regulation was observed in the roots (Table 3). Furthermore, the highest up-regulation of SKP1 was noted in stem for both the varieties under water stress.

**Table 3:** Comparison of relative gene expression level of SKP1 in different plant parts between the two contrasting mungbean cultivars where water was withdrawn for ten consecutive days (With + and −t values, significance at p ≤ 0.05).

Samples	Percent change	p-value	t-value	Result	Significance
Control leaves	46.4	0.023	4.237	Up-regulation	S
Drought leaves	5.93	0.294	0.599	Up-regulation	NS
Control stem	67.87	0.0005	22.556	Up-regulation	S
Drought stem	48.69	0.0015	11.846	Up-regulation	S
Control seedling	78.04	0.0015	16.908	Up-regulation	S
Drought seedling	38.67	0.002	6.445	Up-regulation	S
Control roots	59.44	0.0135	5.775	Up-regulation	S
Drought root	20.39	0.031	−2.575	Down-regulation	S

Note: S- Significant, NS- Not Significant.

### Full length SKP1 ORF cloning from mungbean

**Figure 4:**
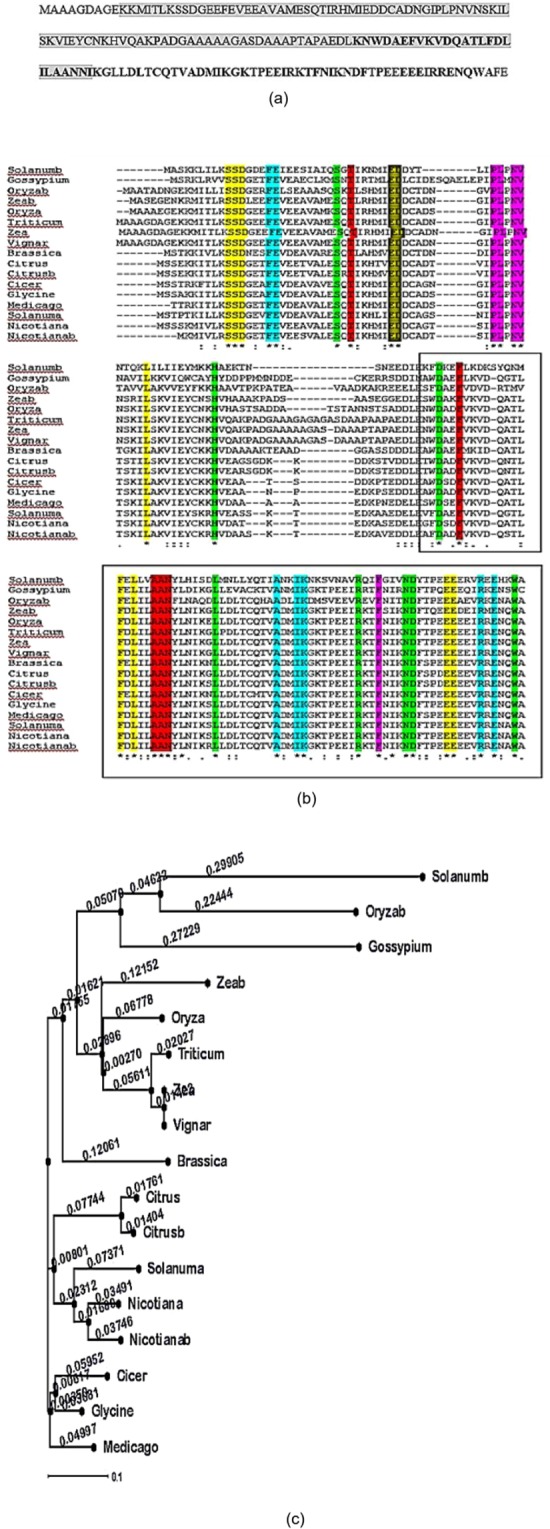
(**a**) VrSKP1 amino acid sequence showing BTB superfamily (98–172) and SKP1 (10–124) superfamily of conserved domains where the former is highlighted in bold, while the later has been shaded with grey. (**b**) CLUSTAL Omega multiple sequence alignment diagram showing predicted amino acid sequence alignments of VrSKP1 with sixteen other SKP1 like genes from various crops and legumes. The conserved amino acid sequences are shaded in grey while the SKP1 domain is boxed. (**c**) Phylogenetic tree constructed using Phyfi software showing VrSKP1 and its inferred evolutionary relationships with SKP1 identified from different crops/legumes.

Full length SKP1 ORF of 550 bp and 114 amino acids was isolated and cloned for the first time from mungbean drought tolerant cultivar Pratap named as VrSKP1 and deposited with the accession number KX881912 to the NCBI nucleotide database. The VrSKP1 contained two conserved domains belonging to super families BTB (98–172 amino acid) and SKP1 (10–124 amino acid) (Fig. 4a). Clustal omega multiple sequence alignment was carried out with various other available SKP1 sequences highlighting the SKP domains (Fig. 4b). VrSKP1 shared the highest percentage of homology with SKP1 identified from *Z*. *mays* (NP_001148633), while the lowest with *Solanum (Solanum tuberosum; XP_015170904*) as depicted in Table 4. The phylogenetic tree constructed with VrSKP1 and other known SKP1 amino acid sequences showed the evolutionary relationships with SKP1 identified from different crops/legumes (Fig. 4c).

**Table 4:** Table depicting similarity match (in percentage) of SKP1 like gene from various crops/legumes viz.

SKP1 amino acid sequence from various crops/legume	Identity % with VrSKP1
*Vigna*	100%
*Zea*	100%
*Triticum a*	96%
*Citrus a*	75%
*Oryz a*	84%
*Citrus b*	74%
*Zea b*	78%
*Medicago*	79%
*Glycine max*	79%
*Cicer arientum*	76%
*Nicotiana b*	76%
*Nicotiana a*	74%
*Brassica*	75%
*Solanum a*	74%
*Oryza b*	59%
*Gossypium hirsutum*	54%
*Solanum b*	45%

Zea a (Z. mays; NP_001148633), Triticum a (Triticum aestivum; AAP79890), Citrus a (Citrus maxima; ACP20181), Oryza a (Oryza minuta; ALA48969), Citrus b (Citrus reticulatae; KT124546), Zea b (Z. mays; NM_001138269), Medicago (Medicago sativa; AF135596), Glycine (Glycine max; KRH76622), Cicer (Cicer arietinum; XP_004512165), Nicotiana b (Nicotiana benthamania; AF494084), Nicotiana a (Nicotiana tabacum; AFB18000), Brassica (Brassica napus; DQ206627), Solanum a (Solanum lycopersicum; ALA48968), Oryza b (Oryza sativa; AAQ01198), Gossypium (Gossypium hirsutum; CDW53975), Triticum b (Triticum aestivum; AIJ50267), Solanum b (S. tuberosum; XP_015170904) with VrSKP1.

### Analysis of sequence and secondary structure

**Figure 5:**
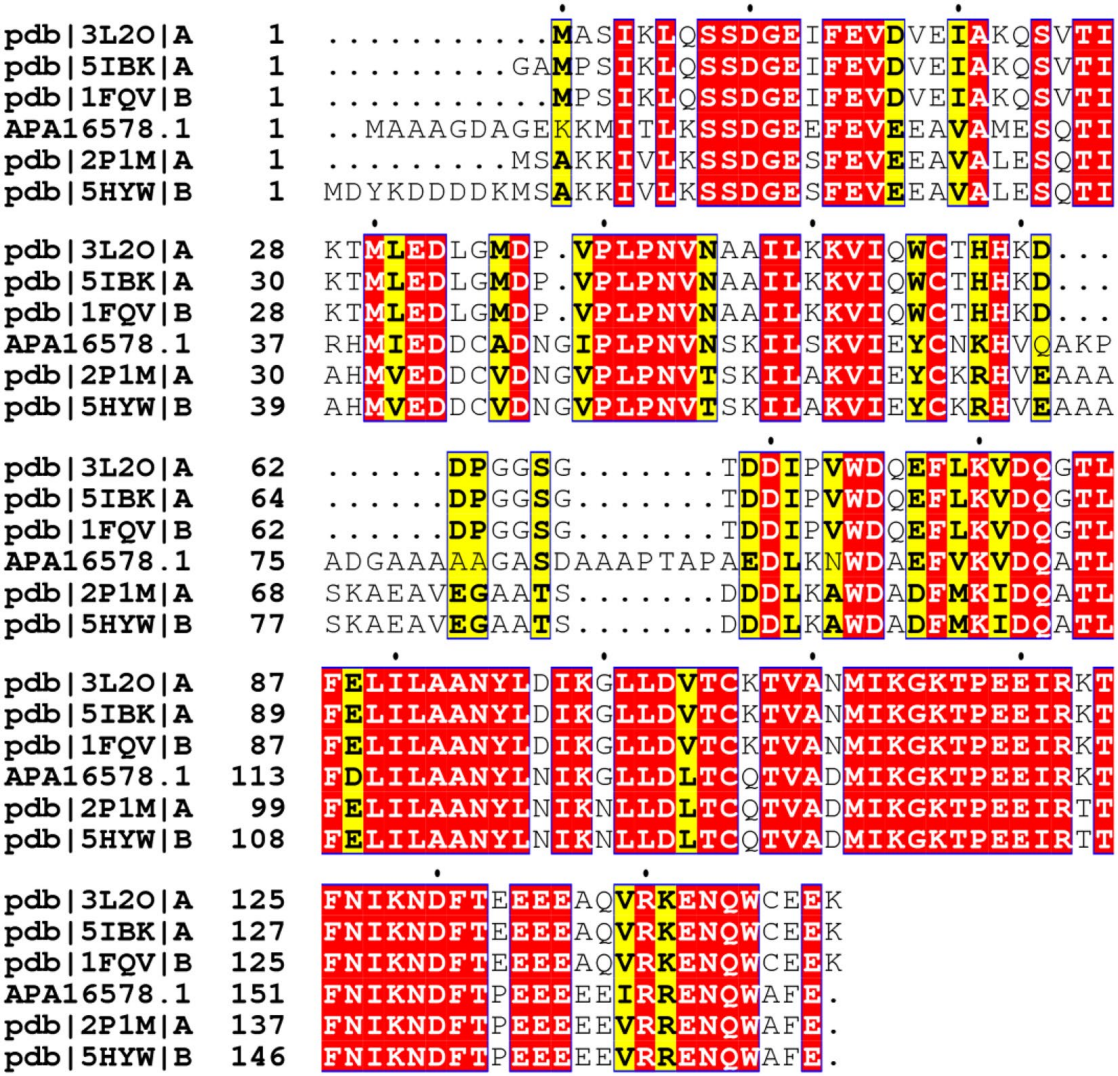
Multiple sequence alignment of VrSKP1 with homologous sequences reported in the protein data bank. The conserved sequences are shown in red background.

**Figure 6:**
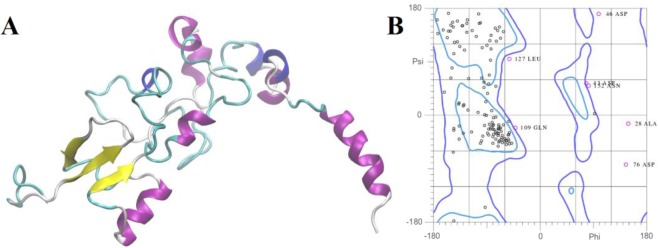
Geometrical and structural analysis of VrSKP1 protein. (**A**) The modelled structure that is stable and has been retrieved from the MD simulation trajectories. The beta sheets are shown as yellow, Alpha-helices as purple, loops as blue and blue as 310 helices. (**B**) Ramachandran plot showing allowed dihedral (phi and psi) angles whereas the pink circles are the residues that are outliers.

The nucleotide sequence of VrSKP1 (GenBank: KX881912.1) was analysed and the nucleotide basic local alignment search tool (blast) searching showed 100% similarity with *Triticum aestivum* S-phase kinase protein. The protein BLAST analysis of the amino acid sequence of the VrSKP1 protein (GenBank: APA16578.1) shows that a large part of the sequence is conserved within the SKPs. The accession number of the homologous proteins having 3D structure in the PDB database is shown in Fig. 5. Following up the structural prediction and validation, the ITASSER server returned 5 models and the best model (Fig. 6A) based on the stereochemical quality was selected for future studies. The Ramachandran plot (Fig. 6B) for the validation of the modelled protein structure provided by Molprobity shows that 79.7% (137/172) of all the residues were in favoured regions, whereas 94.8% (163/172) of all residues were in allowed regions, which allows us to further proceed with the structure. The 9 outliers are Ala28, Asp43, Asp46, Pro52, ASP76, Gln109, Ile124, Leu127 and Asn152. The secondary structure is detailed further in this article.

### MD insights

**Figure 7:**
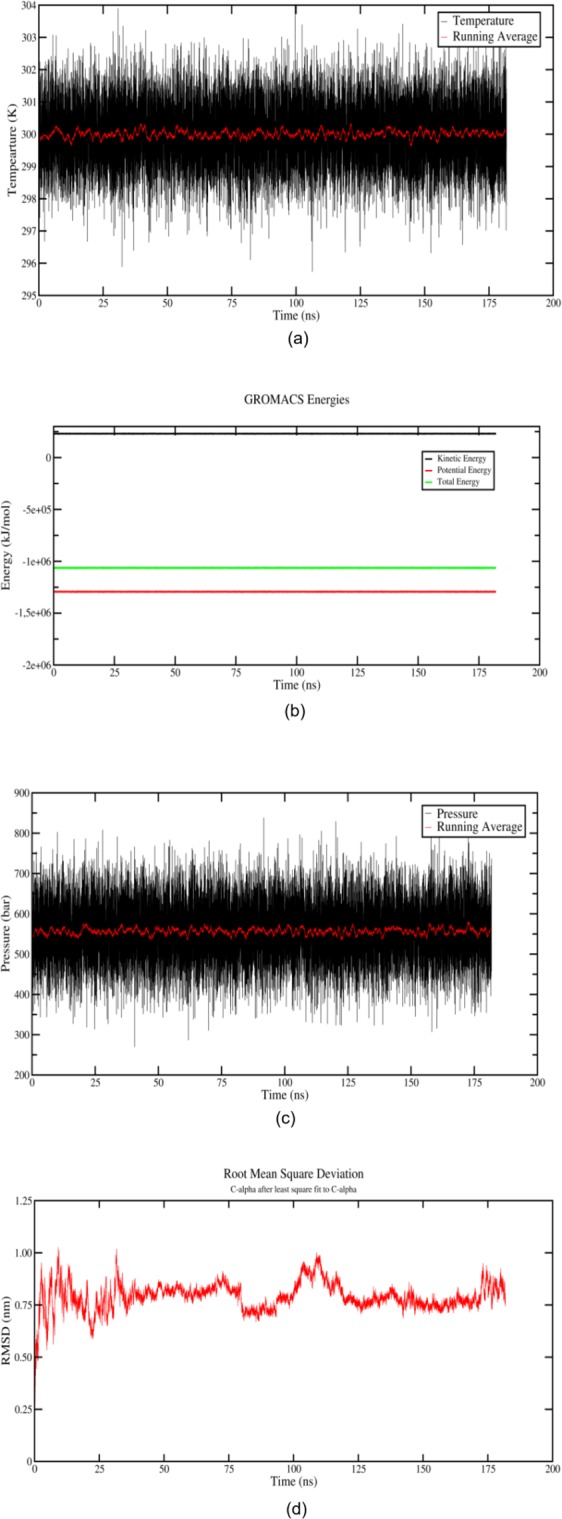
Equilibration properties of VrSKP1 as a function of time for 180 ns. (**a**) Temperature of VrSKP1 protein system throughout the simulation time period. (**b**) Potential, Kinetic and Total energy as a function of time. (**c**) Pressure of the system. (**d**) RMSD variations after C-Alpha has been least square fitted with C-Alpha atoms shown as a function of time.

The temperature plot (Fig. 7a) of the VrSKP1 protein system has been observed with respect to time and has been ensured that the system has safely reached 300 K, the running average of the temperature supports that it has settled around 300 K. To cross verify the system’s stability several test (computational) has been done where the potential, kinetic, total energy and the Root Mean Square Deviation (RMSD) has been plotted as a function of time (Fig. 7b). With a very negligible fluctuations the energy of the system has attained a constant value which proves that the modelled structure has attained stability during the course of simulation. The RMSD which is a measure used to quantify the equilibration of the system (Fig. 7d) has been done after least square fitting the C-Alpha backbone of the configuration of the whole simulation with a reference structure. The reference structure is generally the starting structure of the simulation. The analysis based on RMSD of the C-Alpha backbone showed configurational changes within starting few ns of the simulation and then the system started converging and attains a stable configuration after 50 ns. The stability of the system can be confirmed as the fluctuations are observed as the maximum of 0.99 ns and a minimum of 0.68 ns. As discussed earlier in the materials and method, the system’s pressure has to be calibrated to 560 bar to mimic the density of pure water. It has been shown by the running average of the pressure (Fig. 7c) that it has well settled around 560 ± 10 bar, which again supports the fact that the predicted structure is well stabilized and further can be studied for characterization of the structure.

### Essential dynamics and free energy landscape

**Figure 8:**
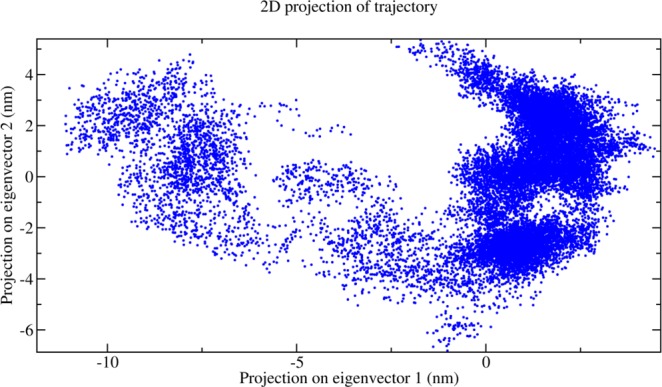
The figure shows the distributions of conformations of VrSKP1 protein projected on to the first two principal components.

**Figure 9:**
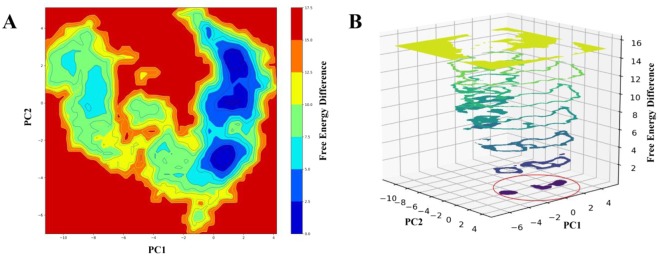
FEL values for the VrSKP1 modelled protein. (**A**) The FEL value has been constructed as a function of projections of the MD trajectories on to their own first (PC1) and second (PC2) eigenvectors. The colour bar represents KJ/mol. (**B**) The 3D contour graph of the FEL has been shown, the red encircle shows the minima of the three clusters that has the lowest energy configurations.

We performed an ED on the MD simulation trajectories to provide an overview of the structural ensembles. This analysis revealed, however, that the ensembles generated by the OPLS-AA force-field sampled a larger region of conformational space. Major confirmation between a few distinct conformational states are expected to be reasonably well described by projections onto the first two principal components shown in Fig. 8. These analyses show that the ensemble has a structural drift and falls roughly into three different clusters of points, which provides the structural basis of the modelled structure. In particular to support this behaviour of the protein structure and to make the conformational differences of the clusters clearer, we have done FEL calculations. The Gibbs free energy differences of the conformational plotted as shown in Fig. 9A,B. It is now very appealing regarding the confirmation of having three major free energy well/basin in the global free energy minimum region, indicating three stable conformational state resided within the well. The lowest energy confirmations are plotted as blue in Fig. 9A and we can clearly observe the three clusters from the 3D contour plot shown in Fig. 9B which supports our prediction. So, we have extracted the most stable structural information from the MD trajectory for the structural characterization which is discussed in the next section. The FEL values and free-energy profiles constructed from our VrSKP1 simulations may have been completed due to the limited sampling time. However, such free-energy calculations are still useful for characterizing the thermodynamics and kinetics of the VrSKP1 protein, which can be taken as further studies.

### Secondary structural prediction and analysis

**Figure 10:**
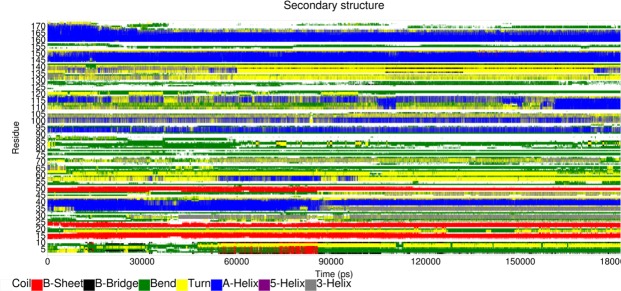
Secondary structure analysis of VrSKP1 has been shown using the DSSP algorithm. The colour code representing the structures has been shown on the right of the graph.

For the first time we report about the 3D structure of VrSKP1 protein predicted after stringent studies. The structure is composed of Beta sheets, Alpha-helix, coils and turns. The Beta sheet mainly includes residues 13–16, 23–25 and 48–50. The Alpha-helix includes regions from 34–42, 64–66, 91–95, 98–103, 114–118, 138–140, 144–151 and 159–171. The main turn regions are 2–8, 26–31, 52–63, 67–73, 77–88, 104–106, 108–113, 119–123, 129–137 and 155–158. The rest of the regions are coil. To analyse the structural changes in the secondary structure as a function of time, the database of secondary structure in proteins (DSSP) software tool was applied. Figure 10 shows that the residue numbers ranging from 91–103 undergoes structural transitions and fluctuates to Alpha-helices to 3_10_ helices.

### Transient expression by GUS assay

**Figure 11:**
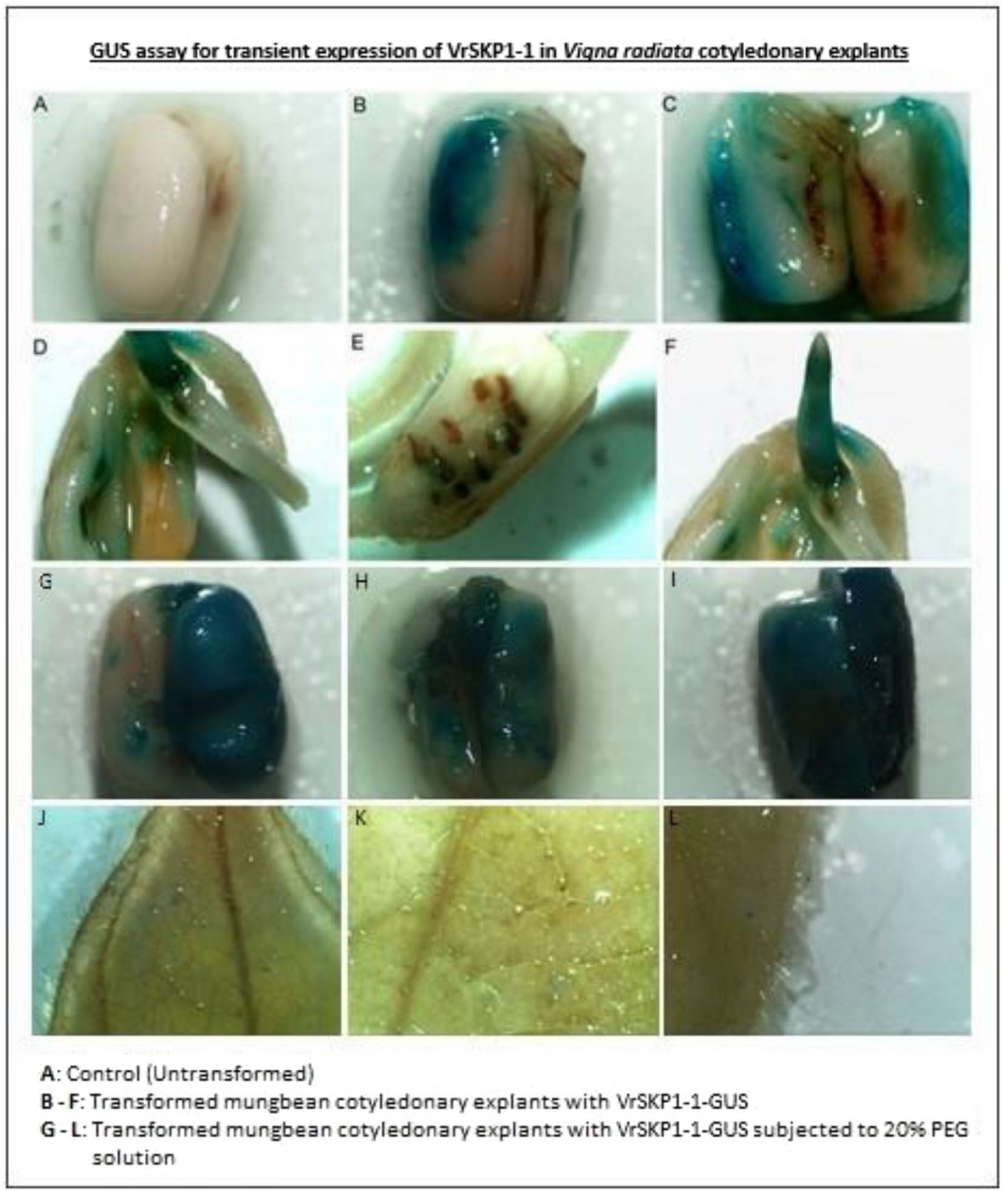
GUS assay showed transient expression of VrSKP1-1 in *V*. *radiata* cotyledonary explants showing insoluble blue colour produced after incubation both under control as well as under drought stress.

The construction of a recombinant plasmid was completed by stacking of a SKP1-1 gene from mungbean before GUS reporter gene in the binary vector pBi121 and confirmed by PCR analysis. Further, transient expression of VrSKP1-1-GUS in *Vigna radiata* was confirmed by GUS assay in putative transgenic mungbean explants. Blue colouration due to oxidation of chloro-bromoindigo of X-Gluc (5-bromo-4-chloro-3-indolyl-β-glucuronidase) was captured with confocal microscope in GUS assay and was assumed as confirmation of SKP1-1 in transgenic mungbean explants (Fig. 11). Higher intensity in blue colouration was also observed in the mungbean cotyledonary explants when exposed to 20% PEG solution. This confirms expression of VrSKP1-1-GUS in response to water stress.

## Discussion

Towards isolation and cloning of full length SKP1 homologue from mungbean, we carried out a detailed transcript expression profiling in vegetative tissues under control as well as under drought treatments in two contrasting mungbean cultivars and further isolated a full length SKP1 ORF.

The study initiated with analyses of some selected physio-biochemical indices in seven mungbean cultivars. For all the studied biochemical parameters, significant differences were observed between the cultivars, treatments, and their interaction (Table 1).

The lowest reduction of leaf RWC recorded in cultivar V7 (Pratap), implied its higher tolerance to water stress. Whereas, the significant decrease of RWC in cultivar V3 (IPM-99-125) illustrated its higher susceptibility by maintaining lesser leaf water under drought. Several researches have also indicated reduced RWC of leaf as one of the early symptoms under drought stress^23^. This decrease in RWC during drought can also be related to the loss of vitality and vigour of the drought treated plant. Researchers have also documented chlorophyll degradation under water stress due to increased production of reactive oxygen species (ROS) like O_2_ and H_2_O_2_ resulting in lipid peroxidation^24^. The lowest value of chlorophyll in cultivar V3 (IPM-99-125) and the highest in cultivar V7 (Pratap) clearly demonstrates their different degrees of drought tolerance. The highest accumulation of sugar and proline in cultivar V7 (Pratap) while the lowest in cultivar V3 (IPM-99-125) could be attributed to the fact that proline and sugar accumulation acts as good compatible solute and serves as a defence mechanism to thrive oxidative stress^25^. Starch reduction is another key attribute to understand drought tolerance^26^. The highest reduction of starch in cultivar V3 (IPM 99-125) is well related to its greater susceptibility towards drought while coinciding with the findings that higher sugar accumulation and concomitant reduction of starch under oxidative stress is in fact due to the subsequent degradation of starch^27^. Reduction of MSI is another key indicator to identify the tolerant and susceptible cultivars under water stress^28^. The higher MSI in cultivar V7 (Pratap) clearly indicates its higher drought tolerance as it goes well with several researchers who opined that electrolyte leakage is correlated with drought tolerance^29,30^ which damages the plant cell membranes making it more permeable^31^. Reduced nitrate reductase (NR) activity under drought, which is one of the key enzymes in nitrogen assimilation has always been referred as good indices to study drought tolerance^32^. Though significant differences of NR activity were recorded in all the cultivars under stress, the difference was not that prominent between cultivars V7 (Pratap) and V3 (IPM 99-125) which might be due to the stage of the crop considered where N-requirement is often low.

Transcript induction of SKP1 was observed under drought with higher accumulation of SKP1 transcripts in both the cultivars; however, the maximum increase was recorded in the drought tolerant cultivar Pratap irrespective of the treatments. SKP1, a vital part of SCF complex consists of 4 major components amongst which the F-box proteins *Arabidopsis thaliana* TUBBY-like protein 9 (AtTLP9) and drought tolerance repressor (DOR) were reported to be involved in ABA signalling^33^. SKP1 actively participates in ABA signalling and thereby regulate numerous biological processes amongst which germination, stomatal aperture closure and root growth is worth a mention^34^. Our study revealed higher inducibility of SKP1 in both the cultivars after stress and the higher relative gene expression in the drought tolerant cultivar Pratap, thereby clearly elucidating the vivid and potential role of SKP1 in drought tolerance. However, the higher induction of SKP1 expression in the drought treated roots of the sensitive cultivar IPM 99-125 over Pratap might be due to the fact that osmotic stress has had its major damage to the root system in particular^35^ which would apparently be higher for the sensitive cultivar in consideration.

Furthermore, the sharp up-regulation of mungbean-SKP1 in leaves and stem of the tolerant cultivar after drought could be attributed to its better inducibility in the actively growing tissues as stated in the findings on XERICO which interacts with an E2 ubiquitin-conjugating enzyme and ASK1-interacting F-box protein AtTLP9^36^. Irrespective of the cultivars, the highest up-regulation of SKP1 noted in the stem could be due to its significant role in auxin signalling pathway as UPS is intricately involved in auxin and various other plant hormone perceptions and signalling.

GUS staining is an easy and reliable method to confirm transient gene expression in plant cells and the best available substrate to analyse β-glucuronidase activity in cells, tissues and bacterial cultures is 5-bromo-4-chloro-3-indolyl-β-glucuronidase (X-Gluc). Blue colour precipitation provides confirmation of transient gene expression at the site of enzyme activity due to oxidation of chloro-bromoindigo indicating positive result for the assay. Further profound research on confirmation of protein expression would provide greater insights into genetic engineering and molecular breeding. It would be extrapolated into faster, easier and convenient inclusion of new and complex traits^37^.

To explore the protein flexibility, molecular motion we performed 180 ns of standard MD. Analysis of geometrical properties suggested that the predicted VrSKP1 modelled protein attains a stable confirmation state. ED and FEL analysis reveals the conformational flexibility with the stable conformational states of the proteins thus extracting the stable confirmation from the MD trajectory also revealing the structural details of the protein.

Our study reports that in mungbean SKP1 and SCF Ubiquitin ligases play critical role in drought tolerance. Further functional characterisation of the gene in mungbean as well as in its wild relatives will reveal its specific role in drought sustenance. The study also reports the first isolation and cloning of SKP1 as a potential candidate gene in drought tolerance from mungbean after an extensive transcript expression profiling in contrasting mungbean cultivars. Further functional characterisation of the gene in mungbean as well as in its wild relatives will reveal its specific role in drought tolerance. Our results also provide affirmation of the GUS-fused chimeric gene construct and its utility in transient GUS assay in both Agrobacterium and plant system and can thereby be recommended to identify positive transgenic plants. This can reduce overall time, labour intensity and resource utilisation of the research process by eliminating negative plants as soon as possible.

## Conclusions

This is the first documented report so far of SKP1 protein in mungbean (tolerant mungbean cultivar, Pratap) on isolation, cloning, expression in binary vector by GUS assay, characterization, protein modelling, and assessment of the protein by computational methods. The significant up-regulation of SKP1 transcript levels documented for the tolerant mungbean cultivar, Pratap, under drought stress corroborates the role of SKP1 as a potential drought responsive candidate gene from mungbean. The MD simulation study reveals the diversified conformations of the modelled SKP1 protein, extracted from *V*. *radiata* and can be incorporated in future transgenic programs for drought tolerance.

## Materials and Methods

### Plant material and growth conditions

The experiment for screening drought tolerant mungbean cultivars was done with seven cultivars in earthen pots during September to December, 2014 in Tezpur University, located at north bank plain zone of Assam (26°14′ and 92°50′), Tezpur, India. To avoid rainfall, earthen pots were kept under a temporary rain shed constructed with polyvinyl chloride (PVC) film of about 0.15 mm thickness and 85% transmittance. Seeds of mungbean cultivars namely, PDM54 (V1), PDM39 (V2), IPM 99-125 (V3), PDM11 (V4), IPM 2-14 (V5), IPM 2-3 (V6) were collected from Indian Institute of Pulses Research (IIPR), Kanpur, India and Pratap (V7) from Regional Agricultural Research Station (RARS), Nagaon, Assam, India. The experiment was done in completely randomized block design with four replications. Regular watering was done in the control plants, while watering was withdrawn in the treated plants after 21 days of germination for 10 consecutive days.

### Physiological and biochemical analyses

Analysis of some already known physiological and biochemical marker traits for drought tolerance was carried out in the second leaf from the top during vegetative stage of the crop. Relative leaf water content in leaves of both control and stressed plants were calculated according to the method described by Yang *et al*.^38^ with the following formula:

$${\rm{RWC}}\,( \% )=[({\rm{FW}}-{\rm{DW}})/({\rm{TW}}-{\rm{DW}})]\times 100 \% .$$

Anderson and Boardman’s^39^ method was used to determine the total chlorophyll content of the cultivars. Chlorophyll stability index (CSI) was then calculated using the following formula:

$${\rm{CSI}}\, \% =({\rm{Total}}\,{\rm{Chlorophyll}}\,{\rm{under}}\,{\rm{stress}}/{\rm{Total}}\,{\rm{Chlorophyll}}\,{\rm{under}}\,{\rm{control}})\times 100.$$

Leaf proline content was determined using the method of Bates *et al*.^40^ and were expressed as μmol g^−1^ fresh weight. Modified method of Jaworski^41^ was utilized to determine nitrate reductase activity. Membrane Stability Index (MSI) was calculated using a method given by Premchandra *et al*.^42^ and as modified by Sairam^43^ using the formula:

$${\rm{MSI}}\,( \% )=[1-({\rm{C}}1/{\rm{C}}2)]\times 100$$

Anthrone method was used for extraction of soluble sugar and the residue left after alcoholic extraction was taken for the extraction of starch and was estimated by the method of McCready *et al*.^44^.

### Water stress treatments, RNA isolation and transcript expression profiling

Seeds from the tolerant and susceptible mungbean cultivars were subsequently germinated in pots filled with autoclaved soil rite and regularly watered in a controlled growth chamber at a temperature of 24 °C in ICAR-National Research Centre in Plant Biotechnology (NRCPB), Indian Agricultural Research Institute (IARI), Pusa, New Delhi. Another set of plants were maintained simultaneously where watering was curtailed to the 21^st^ day old mungbean plants for another ten days. To investigate the expression of SKP1 irrespective of treatments and cultivars, samples from leaves, stems, roots and whole seedlings at vegetative stage were collected, frozen in liquid nitrogen and then stored at −80 °C for further use.

### RNA isolation

Total RNA was isolated from the leaves, stem and whole seedling with triazol reagent (Invitrogen, Carlsbad, USA), whereas, RNA from roots were isolated using Spectrum kit (Sigma, Mo, USA). Isolated RNAs with RNA loading dye were checked in a 1.2% agarose gel. 1 μg of the samples were then DNAse (Promega) treated after nanodrop spectrophotometric quantification (Thermo Fisher Scientific, Wilmington, USA).

### Semi quantitative reverse transcription PCR

DNAse treated RNA was reverse transcribed with superscript III first-strand cDNA Synthesis Kit (Invitrogen, Carlsbad, USA). For every specific reaction, 50 ng of cDNA was used as template with gene-specific primers for amplification, designed from the available SKP1 sequences from public databases at NCBI (www.ncbi.nlm.nih.gov). RT-PCR reactions were then performed in 50 µl volume of reaction mixture containing 1X green master mix (Takara Bio Inc, Shiga, Japan) and 10 nM of each forward and reverse primer. The whole reaction was incubated in a thermal cycler programme of initial denaturation at 94 °C for 4 min followed by 35 cycles of 94 °C for 45 sec-denaturation, 61.4 °C for 45 sec-annealing, 72 °C for 1 min-extension and a final extension at 72 °C for 10 min (Biometra, Goettingen, Germany). The sequences of primers used were (forward-5′TGGCTGCCAACTACCTGAACA3′ and reverse-5′ACATGTTCACCGACACCACCT3′) for mungbean SKP1 gene amplification. Relative expression level of the target genes was determined semi quantitatively with c-DNA synthesized from leaves, stem, roots and whole seedling, checked in 1.2% gel and was analysed with the aid of Syngene Software, Gene tools (Syngene, UK). For quantification, every sample was triplicated and then analysed. Amplification of actin (forward-5′AGCGAGTCTTCATAGGGCGAT3′ and reverse-5′TAGCTCTGGGTTCGAGTGGCA3**′**) was used as an internal control to ensure equal amounts of cDNA and relative quantification.

### Full length SKP1 ORF cloning from mungbean

The full length ORF of SKP-1 was amplified from mungbean cultivar Pratap, using the primers forward-5′ACCACTGACCGAAGATGTTCACCA3′- and reverse-5′GAGCGATGGCGGCCGCGGGAGAC3′. The sequence was cloned in pGMT vector, confirmed by colony PCR and restriction digestion followed by Senger sequencing (AgriGenom, Cochin, India). The sequence homology was carried out with NCBI (www.ncbi.nlm.nih.gov) tools and verified and deposited in gene bank. Multiple sequence alignment with publicly available SKP1 amino acid sequences were carried out using Clustal omega (http://www.ebi.ac.uk/Tools/msa/clustalo/). Phylogenetic tree was drawn using PhyFi (http://cgi-www.daimi.au.dk/cgi-chili/phyfi/go).

Another SKP1 named as SKP1-1(=400 bp), coding sequence was also amplified using primers forward-5′GGTCTAGAATGGCGGCCGCGGGAG3′ and reverse-5′CGCGGATCCCTACTCAAAGGCCCTCTGG3′. The sequence was cloned in pGMT vector, confirmed by colony PCR and restriction digestion followed by Senger sequencing (AgriGenom, Cochin, India).

### Molecular protein modelling and structural refinement

The nucleotide sequence of VrSKP1 gene was subjected to nucleotide Basic Local Alignment Search Tool (BLASTn), it was found to be 100% similar with a mRNA for putative SKP1 protein from *T*. *aestivum* (accession no. AJ577364). Confirming the gene, the nucleotide sequence was submitted to NCBI data bank (accession no. KX881912). The gene sequence was then translated using “Expasy Translate” tool to deduce the protein sequence. Then, the accession no. APA16578.1 of VrSKP1 protein was subjected to BLASTp choosing database option as Protein Data Bank (PDB) proteins. The FASTA sequence of 5 best hits were taken and subjected to Multiple Sequence Analysis (MSA). The accession no. of the respective hits are mentioned in the Fig. 6. The MSA was done in MUSCLE^45^ and ESPript3.0^46^ was used to interpret the MSA results. In order to determine the protein structure, the amino acid sequence was submitted to ITASSER^47^ for protein modelling. Molprobity^48^ validated the modelled structure by plotting the Ramachandran Plot.

### Molecular dynamics (MD) simulation

MD simulation was carried on the modelled structure of the protein (VrSKP1) for 180 ns. Gromacs 5.1.4 software package^49^ has been used with the OPLS-AA force-field. The whole system was then solvated using simple point charge (SPC) water model in a cubic box and 16 Na^+^ counter ions are added to neutralize the system. The system was relaxed using steepest descent algorithm with a time scale of 1 femtosecond. The temperature of the system was maintained at 300 K by the isokinetic temperature coupling, the simulation isobar identified by the density (33.321 molecules/nm^3^) of the reference pure SPC box (i.e. the experimental liquid water density at about 300 K) we utilized to mimic the typical liquid water conditions for inserting the protein molecule into the solvent, corresponds within our simulation conditions to approx. 560 bar instead of the experimental approx. 1 bar^50^. The distance between the closest atoms of the protein and the water box’s wall was set to 1.4 nm. Using periodic boundary conditions 180 ns of simulation was performed in the isothermal-isochoric ensemble (NVT)^51^. All the bonds were constrained using the LINCS algorithm^52^ with a cut-off radius of 1.3 nm for short range interaction. The Particle Mesh Ewald method^53^ was used to compute the long-range interactions with grid search and cut-off radii of 1.3 nm. The secondary structure content along the trajectory was computed according to the DSSP algorithm^54^. The analysis of the trajectory is done using Grace software for plotting the RMSD, temperature, energy, pressure graphs.

### Essential dynamics (ED) and free energy landscaping (FEL)

To elucidate the structure and extract larger amplitude motions observed in the MD trajectories of VrSKP1, ED^55^ was done. A set of eigenvectors and corresponding eigenvalues were elucidated from the diagonalized covariance matrix built obtained from the atomic fluctuations of the MD trajectory. The eigenvectors have all the information regarding the directions of the system in the conformational space representing the collective motions of all the atoms along those directions. The eigenvalues represent the mean square fluctuations (MSF) of atoms along corresponding eigenvectors. In this study, the major motion modes are defined by the first two eigenvectors of the essential conformational sub-space showing most significantly large concerted motions of the system. The covariance matrices of C-Alpha atoms for the VrSKP1 MD trajectories were built and diagonalized using the program g_covar within GROMACS software. The g_anaeig program within GROMACS was used for the projection of trajectories onto the eigenvectors. The two components of the two highest eigenvalues of the PCA calculations are taken on the x and y direction respectively for the 2D projection and plotted using Grace. In-house fortran95 codes were used for further conversions of data to be read by sham program of GROMACS for Gibbs free energy landscape (FEL). In-house python codes were used on the outputs of sham to convert the binary files into human readable files. The 3D profiling in the form of graphs for the free-energy differences, contourf 3D graph are done using python codes, matplotlib and numpy in Jupyter-notebook.

### Construction of gene stacked binary vector and mobilization of pBi121/VrSKP1-1-GUS into *Agrobacterium tumefaciens* EHA105

A recombinant plasmid was constructed by recovery and ligation of plasmid pBi121 and SKP1-1 cloned in pGMT vector after restriction-digestion with XbaI and BamHI under the control of CaMV35S sequentially with VrSKP1-1 before GUS as the reporter gene which was confirmed with PCR and restriction digestion in *E*. *coli*. Later, it was mobilized into *Agrobacterium tumefacieans* EHA105 by freeze and thaw method^56^ and named as pBi121/VrSKP1-1-GUS. Single colony picked up from freshly grown *Agrobacterium* cultures carrying vector plasmids pBi121/VrSKP1-1 with appropriate antibiotics viz; kanamycin (50 mg/ L), rifampicin (75 mg/L) was grown in YEM broth at 28 °C for 2–3 days at 220 rpm. The cell culture was harvested and transferred into fresh YEM broth (10–20 ml) along with appropriate antibiotics after the cell reached the exponential (or log) phase and used for plant transformation.

### Mungbean transformation using apical meristem *in-planta* method and GUS histochemical assay

Seeds of *V*. *radiate* were surface-sterilized and germinated in wet petri-plates for 2 days. Two sets of plants were made and labelled as C (control-untransformed and control-transformed) and T (treated-transformed). Post-germination, a radical injury was made with a sharp scalpel in the mungbean germinating seeds. *Agrobacterium* suspension culture having VrSKP1-1-GUS under the control of CaMV35S promoter was prepared by centrifuging it at room temperature for 10 minutes at 5000 rpm and further re-suspending the pellet in 10 ml YEM (OD = 0.4–0.6). Agitation of mungbean seeds in the suspension for 2 hrs followed by washing them by cefotaxime was accomplished. After being wet-dried in tissue paper for another 2 days, the seeds were placed back into the original plate. Another set of mungbean seeds were subjected to 3 hours of 20% PEG treatment & then allowed to germinate. After germination, some of the seeds (control-untransformed, control-transformed, treated-20% PEG solution) were utilized for GUS assay while some were directly transferred to soil. Histochemical assays for GUS activity on the germinated seeds, cotyledonary radicle of putative transgenic plants and leaf of one-week old proliferating explants was performed for transient and stable expression of GUS gene. The mungbean cotyledonary explants were collected and submerged in 20 ml of GUS substrate (1M sodium phosphate pH 7/Triton-X 20%/X-Gluc/water). The mungbean seeds were then covered with aluminium foil and placed in dark condition to be incubated for 48 hours at 37 °C. This was then rinsed in 75% alcohol two or three times to remove chlorophyll^57^. The tissues of positive transgenic plants would show insoluble blue colour precipitate of dichloro-dibromo indigo. Transient reporter gene expression was used as an indicator of the expression of SKP1-1 transcripts.

### Statistical analysis

The data obtained from biochemical estimation was analysed by Analysis of Variance (ANOVA) using a statistical computer package Statistix Ver. 8.1. The Duncan multiple range test (*p* ≤ 0.05) was used for mean separation within treatments and cultivars.

To analyse the relative expression levels for a cultivar in a treatment one-way ANOVA was done. In similar grounds, t-test was conducted to compare the relative expression levels of SKP1 in the cultivars within/between the treatments for different plant parts. The differences within treatments and cultivars were estimated using Duncan multiple range test (*p* ≤ 0.05)

## Supplementary information

Supplementary File 1

## Data Availability

All data generated or analysed during this study are included in this published article (and its Supplementary Information Files).

## References

[1] Kilic H, Yagbasanlar T (2010). The effect of drought stress on grain yield, yield components & some quality traits of durum wheat (*Triticum turgidum* ssp. durum) cultivars. Not. Bot. Hort. Agrobot. Cluj..

[2] Singh P, Tiwari A, Singh SP, Asthana RK (2013). Proline biosynthesizing enzymes (glutamate 5-kinase & pyrroline-5-carboxylate reductase) from a model cyanobacterium for desiccation tolerance. Physiol. Mol. Biol. Plants..

[3] Bohnert HJ, Jensen RG (1996). Metabolic engineering for increased salt tolerance-the next step. Aust. J. Plant Physiol..

[4] Czech L, Stöveken N, Bremer E (2016). EctD-mediated biotransformation of the chemical chaperone ectoine into hydroxyectoine & its mechanosensitive channel-independent excretion. Microb. Cell Fact.

[5] Deokar, A. A. *et al*. Comparative analysis of expressed sequence tags (ESTs) between drought-tolerant & susceptible genotypes of chickpea under terminal drought stress. *BMC Plant Biol*. 111–20 (2011). 10.1186/1471-2229-11-70 PMC3110109 21513527

[6] Hiremath PJ (2011). Large-scale transcriptome analysis in chickpea (*Cicer arietinum* L.), an orphan legume crop of the semi-arid tropics of Asia & Africa. Plant Biotechnol. J..

[7] Nayak S (2009). Isolation & sequence analysis of DREB2A homologues in three cereal & two legume species. Plant Sci.

[8] Cortés AJ, This D, Chavarro C, Madriñán S, Blair MW (2012). Nucleotide diversity patterns at the drought-related DREB2 encoding genes in wild & cultivated common bean (*Phaseolus vulgaris* L.). Theor. Appl. Genet.

[9] Manavalan LP (2009). Physiological & molecular approaches to improve drought resistance in soybean. Plant & Cell Physiology.

[10] Iuchi S, Yamaguchi-Shinozaki K, Urao T, Terao T, Shinozaki K (1996). Novel drought-inducible genes in the highly drought-tolerant cowpea: cloning of cDNAs & analysis of the expression of the corresponding genes. Plant Cell Physiol..

[11] Saxena KB (2011). Enhancing the livelihoods of Uttarakhand farmers by introducing pigeon pea cultivation in hilly areas. J. Food Legumes.

[12] Farooq, M. *et al*. Drought Stress in Grain Legumes during Reproduction & Grain Filling. *J*. *Agro*. *Crop Sci*. 1–23 (2016).

[13] Hu D-L (2013). Tang, Identification of cotton SKP1-like gene GhSKP1 & its function in seed germination & taproot growth in tobacco. Can. J. Plant Sci.

[14] Hellmann H, Estelle M (2002). Plant development: regulation by protein degradation. Science.

[15] Chen Y, Chi Y, Meng Q, Wang X, Yu D (2018). GmSK1, an SKP1 homologue in soybean, is involved in the tolerance to salt and drought. Plant Physiol. Biochem..

[16] Dreher K, Callis J (2007). Ubiquitin, hormones & biotic stress in plants. J. Ann. Bot..

[17] Zhang Y (2015). Genome-wide analysis of phylogeny, expression profile and sub-cellular localization of SKP1-Like genes in wild tomato. Plant Sci..

[18] Borges A, Tsai SM, Caldas DG (2012). Validation of reference genes for RT-qPCR normalization in common bean during biotic & abiotic stresses. Plant Cell Rep..

[19] Jefferson RA (1987). Assaying chimeric genes in plants: the GUS gene fusion system. Plant Mol. Biol. Rep..

[20] Song JB (2015). The F-box family genes as key elements in response to salt, heavy mental, & drought stresses in *Medicago truncatula*. Funct. Integr. Genomics.

[21] Hao Q (2016). Overexpression of PSK1, a SKP1-like gene homologue, from *Paeonia suffruticosa*, confers salinity tolerance in Arabidopsis. Plant Cell Rep..

[22] Liu A (2015). Zhu, GsSKP21, a *Glycine soja* S-phase kinase-associated protein, mediates the regulation of plant alkaline tolerance & ABA sensitivity. Plant Mol. Biol.

[23] Halder KP, Burrage SW (2003). Drought stress effects on water relations of rice grown in nutrient film technique. Pakistan J. Biol. Sci.

[24] Alaei Y (2011). The effect of amino acids on leaf chlorophyll content in bread wheat genotypes under drought stress conditions. Middle-East J. Sci. Res..

[25] Yoshiba Y, Kiyosue T, Nakashima K, Yamaguchi-Shinozaki K, Shinozaki K (1997). Regulation of levels of proline as an osmolyte in plants under water stress. Plant Cell Physiol.

[26] Patakas A, Nikolaou N, Zioziou E, Radoglou K, Noitsakis B (2002). The role of organic solute & ion accumulation in osmotic adjustment in drought stressed grapevines. Plant Sci.

[27] Mohammadkhani N, Heidari R (2008). Drought induced accumulation of soluble sugars & proline in two maize varieties. W Applied Sci J.

[28] Rudolph AS (1986). Crowe, Effects of three stabilising agents - proline, betaine & trehalose -on membrane phospholipids. Arch. Biochem. Biophys.

[29] Martin U, Pallardy SG, Bahari ZA (1987). Dehydration tolerance of leaf tissues of six woody angiosperm species. Physiol. Plant.

[30] Vasquez-Tello A, Zuily-Fodil Y, Pham Thi AT, Vieira da Silva JB (1990). Leakages & soluble sugar content as physiological tests for screening resistance to water stress in *Phaseolus* & *Vigna* species. J. Exp. Bot..

[31] Senaratana T, Kersi BD (1983). Characterization of solute efflux from dehydration injured soybean *(Glycine maxl*, Merr.). seeds. Plant Physiol.

[32] Foyer CH, Valadier MH, Migge A, Becker TW (1998). Drought-induced effects on nitrate reductase activity & mRNA & on the coordination of nitrogen & carbon metabolism in maize leaves. Plant Physiol.

[33] Lai C-P (2004). Molecular analyses of the Arabidopsis TUBBY-like protein gene family. Plant Physiol..

[34] Li C (2011). SKP1 is involved in abscisic acid signalling to regulate seed germination, stomatal opening & root growth in *Arabidopsis thaliana*. Plant Cell Environ..

[35] Valentovič P, Luxová M, Kolarovič L, Gašparíková O (2006). Effect of osmotic stress on compatible solutes content, membrane stability & water relations in two maize cultivars. Plant Soil Environ..

[36] Ko JH, Yang SH, Han KH (2006). Up-regulation of an Arabidopsis RING-H2 gene, XERICO, confers drought tolerance through increased abscisic acid biosynthesis. Plant J.

[37] Nandy S, Zhao S, Pathak B, Muthusamy M, Srivastava V (2015). Gene stacking in plant cell using recombinases for gene integration & nucleases for marker gene deletion. BMC Biotec..

[38] Yang JY, Zheng W, Tian Y, Wu Y, Zhou DW (2011). Effects of various mixed salt-alkaline stresses on growth, photosynthesis, & photosynthetic pigment concentrations of *Medicago ruthenica* seedlings. Photosynthetica.

[39] Anderson JM, Boardman NK (1964). Studies on the greening of dark grown bean plants. II. Development of photochemical activity. Biol Sci..

[40] Bates LS, Waldren RP, Teare ID (1973). Rapid determination of free proline water stress studies. Plant Soil.

[41] Jaworski EG (1971). Nitrate reductase assay in intact plant tissues. Biochem. Biophys. Res. Commun.

[42] Premachandra GS, Saneoka H, Ogata S (1990). Cell membrane stability, an indicator of drought tolerance as affected by applied nitrogen in soybean. J. Agric. Sci. Camb.

[43] Sairam RK (1994). Effect of homo brassinalide application on plant metabolism & grain yield under irrigated & moisture stress conditions of two wheat varieties. Plant Growth Regul..

[44] Cready MM, Guggolz J, Silviera V, Owens HS (1950). Determination of starch & amylose in vegetables. Anal. Chem..

[45] Edgar RC (2004). MUSCLE: multiple sequence alignment with high accuracy & high throughput. Nucleic Acids Res..

[46] Robert X, Gouet P (2014). Deciphering key features in protein structures with the new ENDscript server. Nucleic Acids Res..

[47] Yang J (2015). The I-TASSER Suite: protein structure & function prediction. Nature methods..

[48] Chen VB (2010). MolProbity: all-atom structure validation for macromolecular crystallography. Acta Crystallographica Section D: Biol Crystallogr..

[49] Berendsen HJC, van der Spoel D, van Drunen R (1995). Gromacs: a message-passing parallel molecular dynamics implementation. Computer Physics Comm..

[50] Galdo SD, Paolo M, D’Abramo M, Amadei A (2015). *In-silico* characterization of protein partial molecular volumes & hydration shells. Phys Chem Chem Phys..

[51] Dunning TH (1989). Gaussian basis sets for use in correlated molecular calculations. I. The atoms boron through neon & hydrogen. J Chem Phys..

[52] Hess B (1997). LINCS: a linear constraint solver for molecular simulations. J Comput Chem..

[53] Darden T, York D, Pedersen L (1993). Particle mesh Ewald: An N⋅ log (N) method for Ewald sums in large systems. J Chem Phys..

[54] Kabsch W, Sander C (1983). Dictionary of protein secondary structure: pattern recognition of hydrogen‐bonded & geometrical features. Biopolymers..

[55] Amadei A, Linssen ABM, Berendsen HJC (1993). Essential dynamics of proteins. Proteins: Structure, Function, & Bioinformatics..

[56] Hofgen R, Willmitzer L (1988). Storage of competent cells for *Agrobacterium* transformation. Nucleic Acids Res..

[57] Jefferson RA, Kavanagh TA, Bevan MW (1987). GUS fusions: Beta-glucuronidase as a sensitive and versatile gene fusion marker in higher plants. The EMBO Journal..

