# Author Correction: Transcript expression profiling in two contrasting cultivars and molecular cloning of a SKP-1 like gene, a component of SCF-ubiquitin proteasome system from mungbean *Vigna radiate* L.

**DOI:** 10.1038/s41598-020-60490-9

**Published:** 2020-02-19

**Authors:** Nandita Bharadwaj, Sharmistha Barthakur, Akash Deep Biswas, Monoj Kumar Das, Manpreet Kour, Anand Ramteke, Nirmali Gogoi

**Affiliations:** 1 Department of Environmental Science, Tezpur University, Tezpur, 784028 Assam India; 2 ICAR-National Research Centre on Plant Biotechnology, Pusa Campus, New Delhi 110012 India; 3 Department of Chemistry, Scuola Normale Superiore di Pisa, Piazza dei Cavalieri, 7, Pisa, 56126 Italy; 4 Department of Molecular Biology and Biotechnology, Tezpur University, Tezpur, Assam 784028 India

Correction to: *Scientific Reports* 10.1038/s41598-019-44034-4, published online 30 May 2019

The original version of this Article contained an error in the affiliation for Akash Deep Biswas, which was incorrectly given as ‘Scuola Normale Superiore di Pisa, Piazza dei Cavalieri 7, Pisa, 56126, Italy’. The correct affiliation is listed below:

Department of Chemistry, Scuola Normale Superiore di Pisa, Piazza dei Cavalieri, 7, Pisa, 56126 Italy.

This error has now been corrected in the HTML and PDF versions of the Article, and in the accompanying Supplementary Material.

In addition, the Supplementary Material originally accompanying this Article contained a typographical error in the spelling of the author Akash Deep Biswas, which was incorrectly given as Akashdeep Biswas. This error has now been corrected.

