# Supplementary material for: Transcript expression profiling in two contrasting cultivars and molecular cloning of a SKP-1 like gene, a component of SCF-ubiquitin proteasome system from mungbean Vigna radiate L
Source: Sci Rep. 2019 May 30;9:8103. doi: 10.1038/s41598-019-44034-4 (PMC6542820; doi:10.1038/s41598-019-44034-4)
Supplement: Supplementary file 1 [file 41598_2019_44034_MOESM1_ESM.pdf]

Supplementary Information File

Transcript expression profiling in two contrasting cultivars & molecular cloning of a SKP-1 like gene, a component of SCF-ubiquitin proteasome system from mungbean *Vigna radiate* L.

Nandita Bharadwaj[1], Sharmistha Barthakur[2], Akash Deep Biswas[3], Monoj Kumar Das[4], Manpreet Kour[4], Anand Ramteke[4], Nirmali Gogoi[1]

[1] Department of Environmental Science, Tezpur University, Tezpur 784028, Assam, India

[2] ICAR-National Research Centre on Plant Biotechnology, Pusa Campus, New Delhi 110012, India.

[3] Department of Chemistry, Scuola Normale Superiore di Pisa, Piazza dei Cavalieri, 7, Pisa, 56126 Italy

[4] Department of Molecular Biology and Biotechnology, Tezpur University, Tezpur 784028, Assam, India

nanditatezpur123@gmail.com*; sbthakur@yahoo.com; akash.biswas@sns.it; monojkumar1785@gmail.com; brandmk93@gmail.com; anand@tezu.ernet.in; nirmali@tezu.ernet.in;

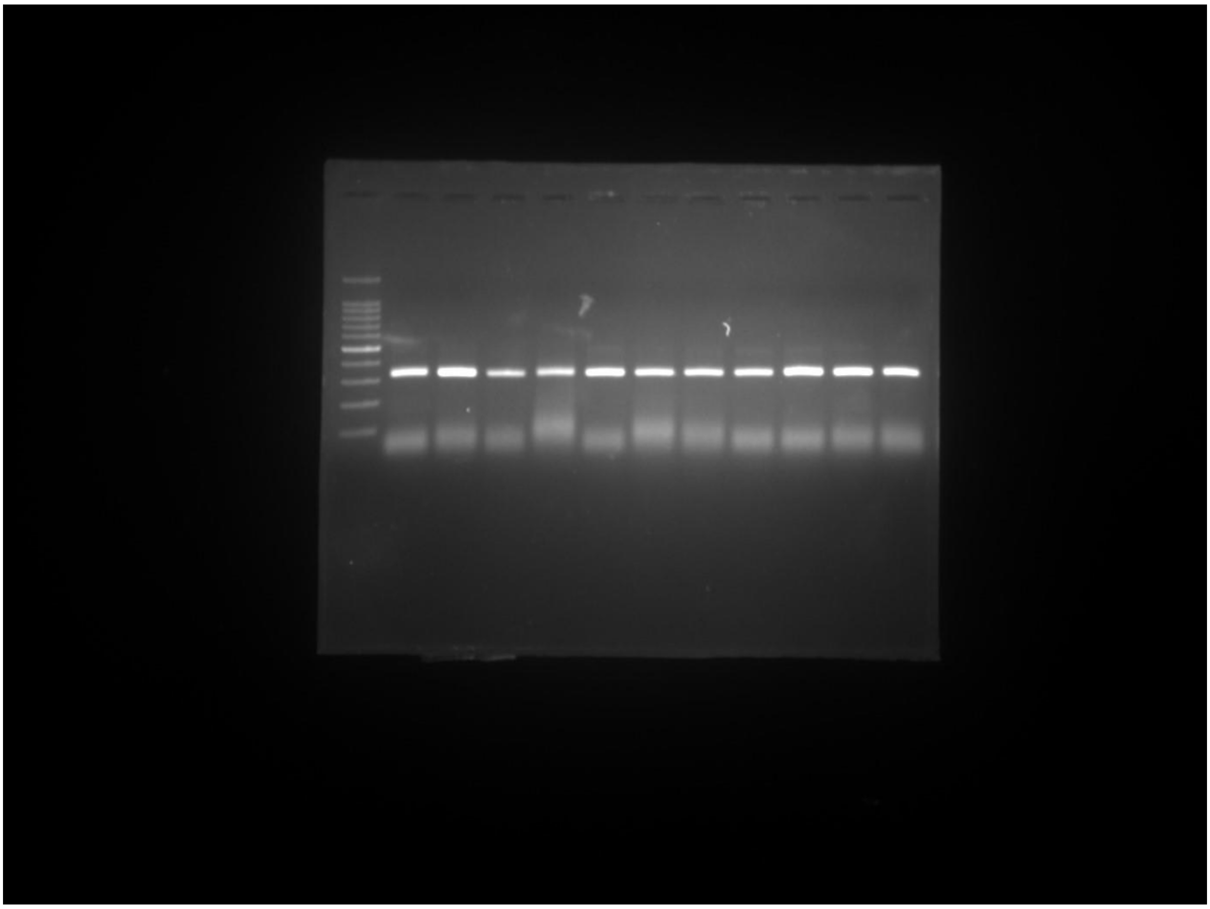

Gel picture-A

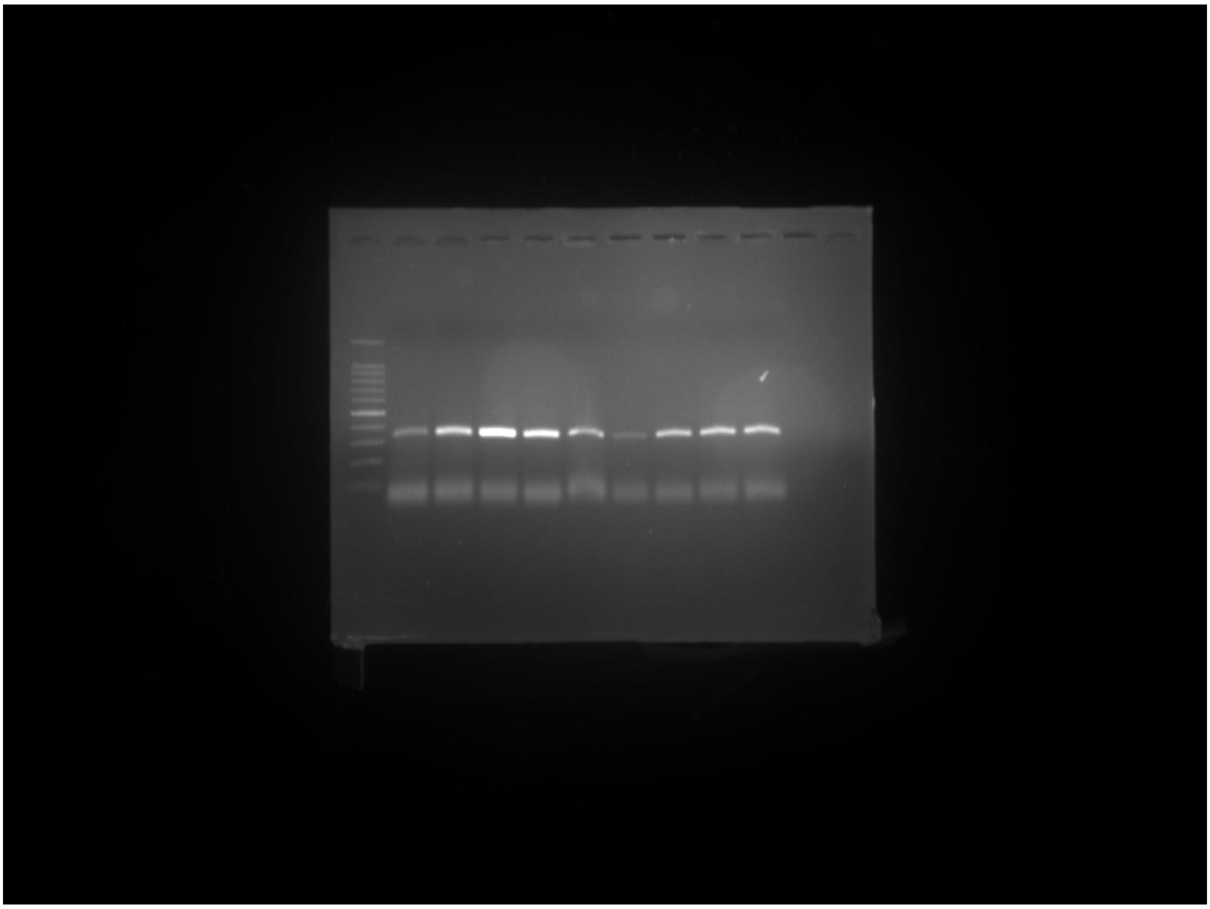

Gel picture-B

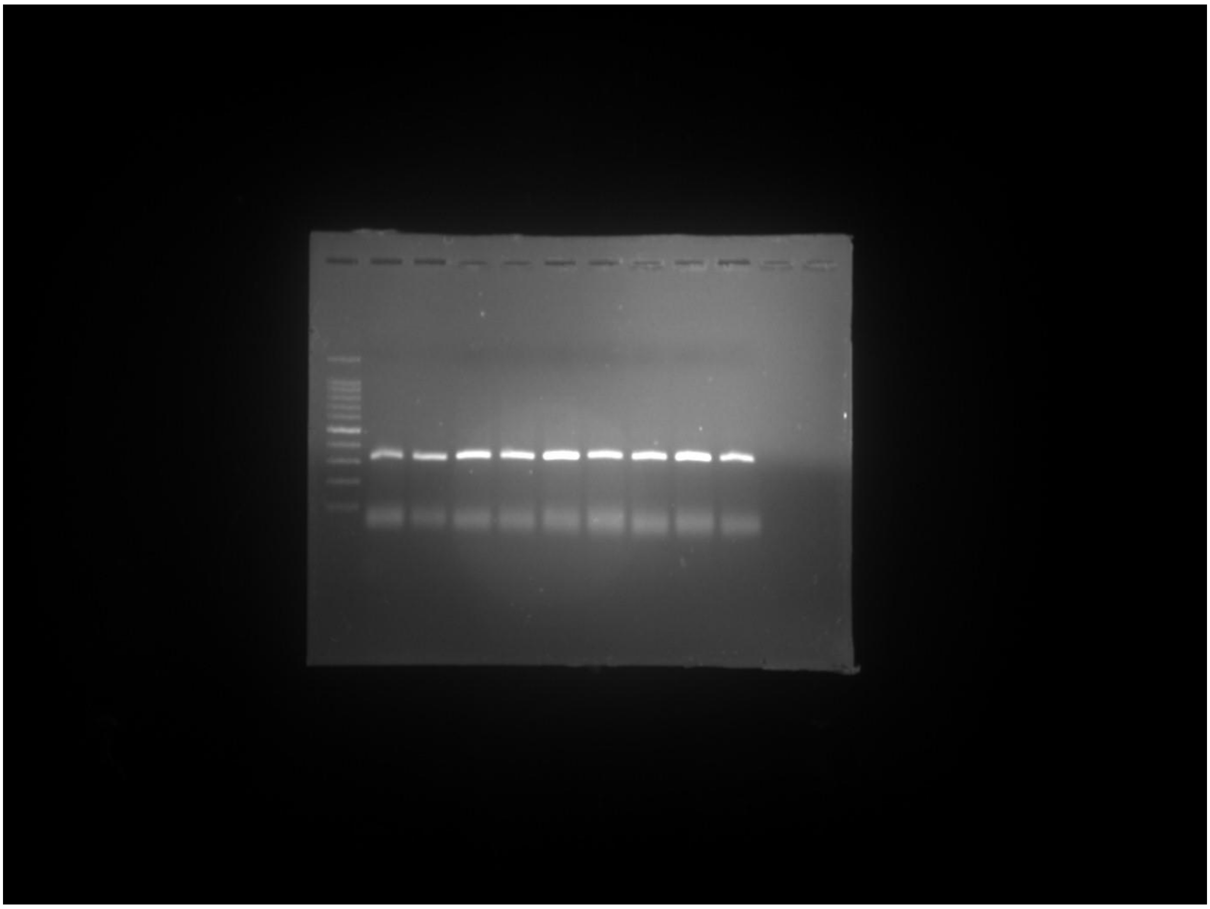

Gel picture-C

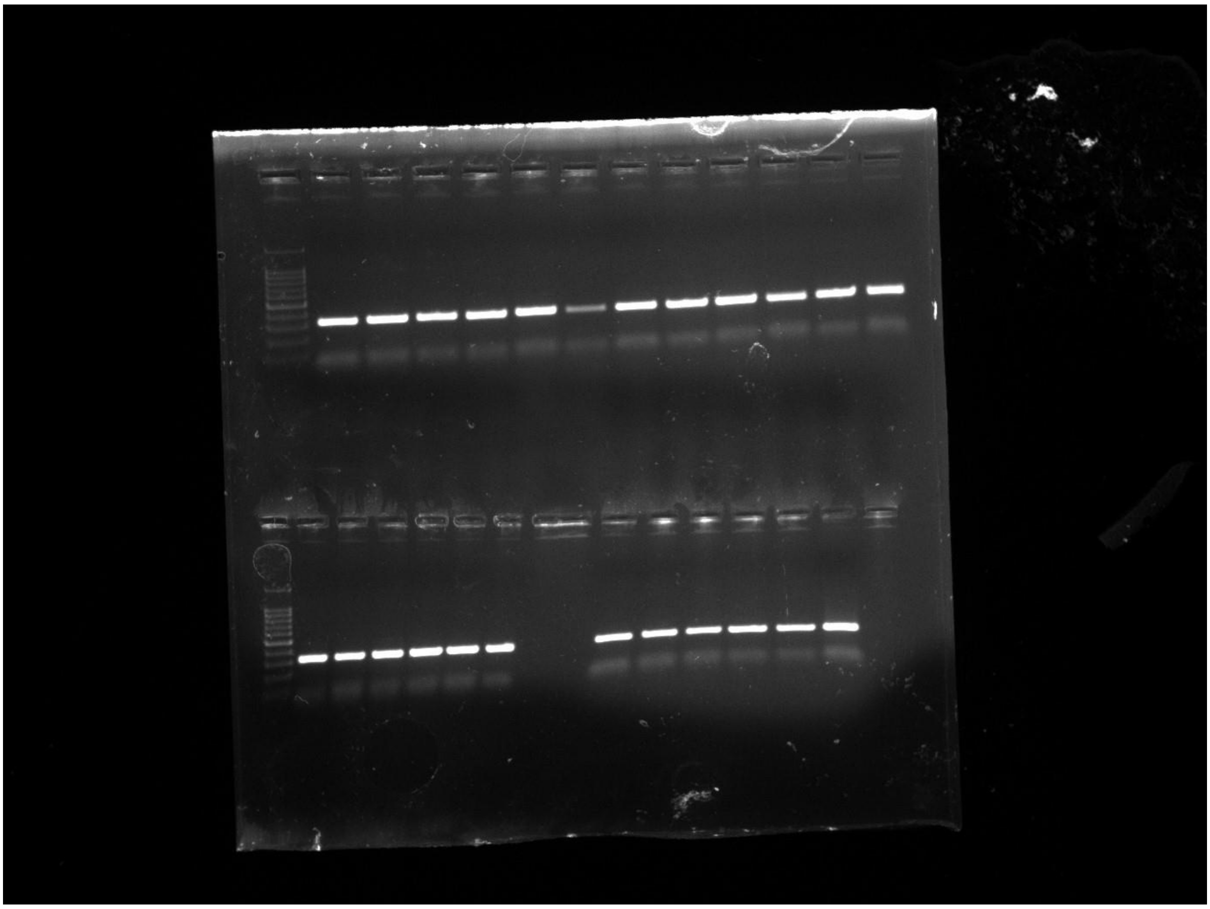

Gel picture-D

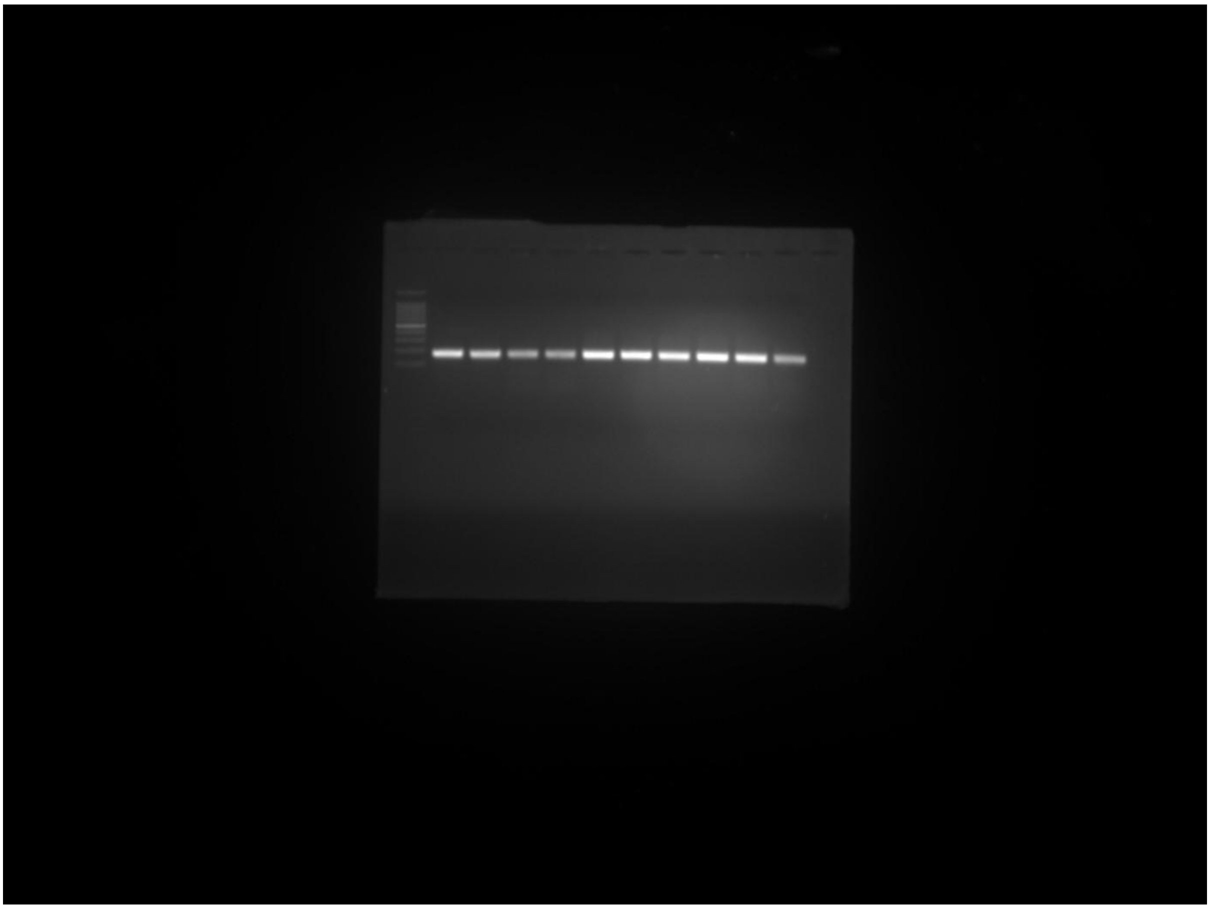

Gel picture-E

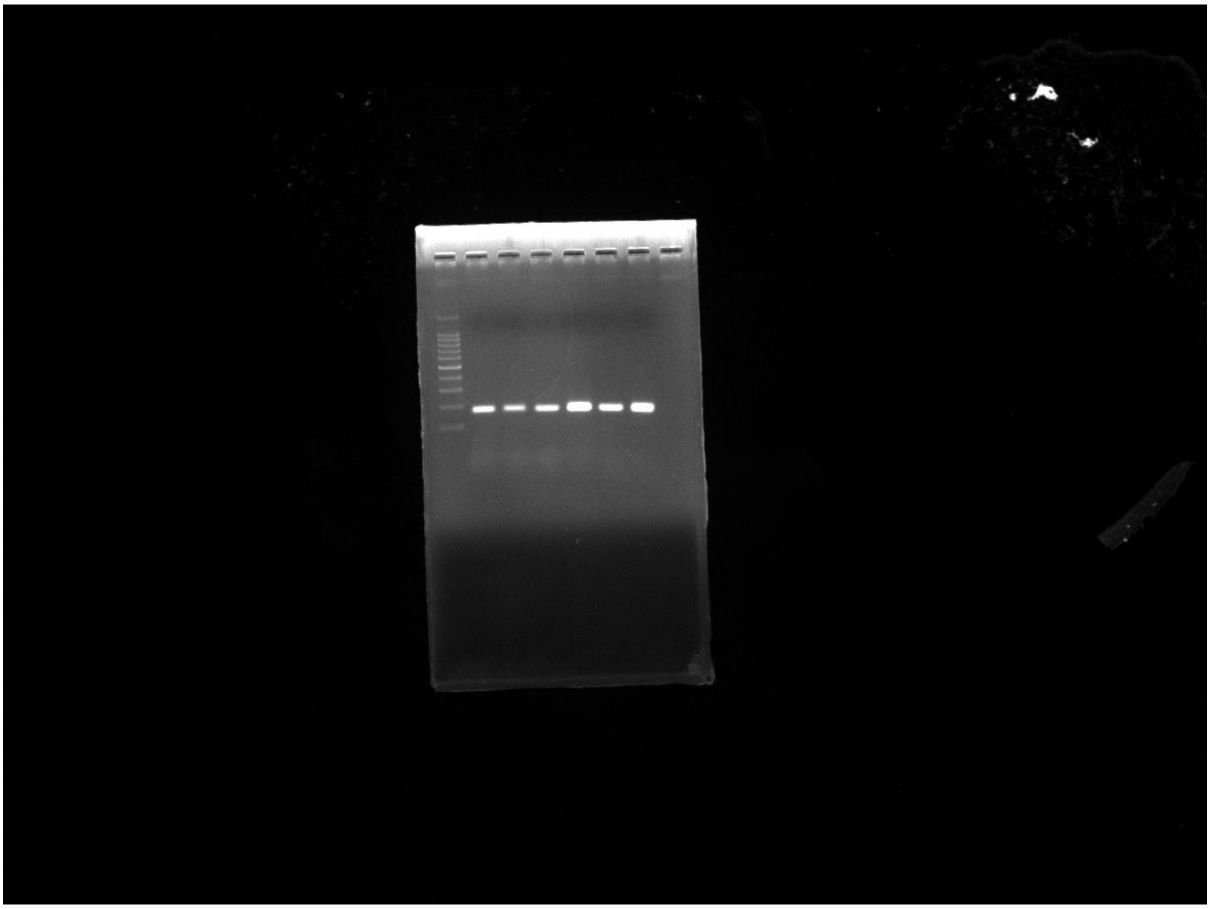

Gel picture-F
